# Preparation of isolated guard cells, containing cell walls, from *Vicia faba*

**DOI:** 10.1371/journal.pone.0299810

**Published:** 2024-03-21

**Authors:** Sara K. Fleetwood, Maya Kleiman, E. Johan Foster

**Affiliations:** 1 Department of Chemical and Biological Engineering, University of British Columbia, Vancouver, British Columbia, Canada; 2 Plant Sciences Institute, Agricultural Research Organization (Volcani Center), Rishon LeZiyyon, Israel; Sri Padmavati Women’s University: Sri Padmavati Mahila Visvavidyalayam, INDIA

## Abstract

Stomatal movement, initiated by specialized epidermal cells known as guard cells (GCs), plays a pivotal role in plant gas exchange and water use efficiency. Despite protocols existing for isolating GCs through proplasting for carrying out biochemical, physiological, and molecular studies, protocals for isolating GCs with their cell walls still intact have been lacking in the literature. In this paper, we introduce a method for the isolation of complete GCs from *Vicia faba* and show their membrane to remain impermeable through propidium iodide staining. This methodology enables further in-depth analyses into the cell wall composition of GCs, facilitating our understanding of structure-function relationship governing reversible actuation within cells.

## Introduction

Guard cells (GCs) play a key physiological role within plants, modulating their transpiration level by balancing CO_2_ uptake with water loss to control photosynthesis through the opening and closing of their central pores, referred to as stomata [1–4]. Additionally, GCs are a model system used to study the signal transduction pathways [5, 6], osmotic properties [7, 8], membrane characterization [9], etc [10]. For these reasons, GCs are a commonly studied type of plant cell. Despite a majority of plants and aerial plant organs (ex. leaves, stems, petioles, primary roots, nectaries, moss, hornworts, lycophytes, ferns) containing GCs [3, 11, 12], with leaves containing the greatest numbers of GCs [13], it remains a challenge studying these cells due to their lack of abundance with respect to other cellular contents within the epidermal tissue of plants.

Currently, GCs are studied directly within tissue (i.e. epidermal peels) [14, 15] or after isolation, however these methods result in contamination or partial loss of the GC’s cellular contents. Specifically, when the epidermis is peeled away from the leaf, contaminates, primarily consisting of chloroplasts and epidermal cells, remain, limiting our ability to study the cellular contents of GCs. For isolation, the most common technique used is protoplasting, in which GCs are isolated through blending, filtering, and enzyme digestion, but no longer contain a cell wall [10]. This technique commonly uses *Vicia faba* leaves due to the ease of separating the epidermis from the mesophyll and it being a model plant of leguminous crops, leading to any new insights furthering our agronomic knowledge of leguminous crops [16]. However, removal of the cell wall during protoplasting has limited our ability to study the structure and chemical composition of GC cell walls. Therefore, it would be advantageous to develop a GC isolation method in which the GC’s cell walls remain intact, with minimal contamination present. Doing so would enable botanists to obtain a global quantitative analysis of GC wall composition in comparison to other cell types and would aid with further investigation into structure-function relationships governing reversible actuation within cells [17].

**Fig 1:**
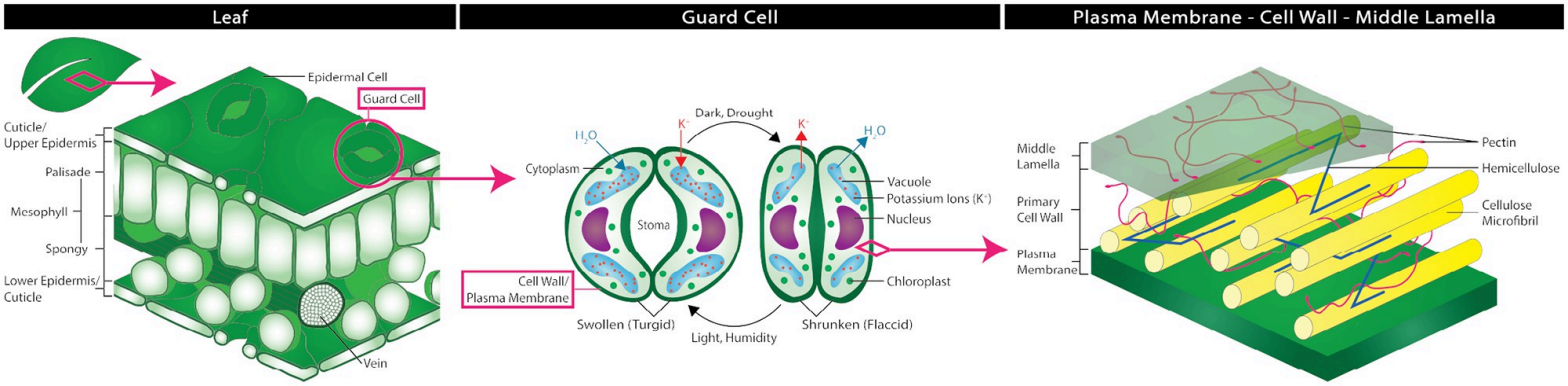
Leaf anatomy. The anatomy of a leaf, including: (left) a leaf cross section, (center) the cellular components of open and closed guard cells, and (right) the cell wall’s composition. The upper and lower epidermis are just below the cuticle and contain guard cells, which open and close to allow gas exchange in and out of the leaf’s mesophyll.

In developing a new procedure for GC isolation that does not remove the cell wall, it is important to have an understanding in how GCs differ from other plant tissues found within leaves. Leaves consist of a spongy mesophyll layer sandwiched between tough upper and lower epidermal layers (Fig 1). The epidermal layers include epidermal cells, subsidary cells, and GCs [1, 18, 19], which are held together by the pectin-rich middle lamella [20, 21]. To enable the repeat opening and closing of GCs, many plant species have developed GCs with higher concentrations of cellulose in comparison to other cell types found within leaves (ex. epidermal cells) [22, 23]. Due to this increased cellulose contents, it is anticipated that through a ‘lighter’ digestion process (i.e. shorter digestion time at a lower enzyme concentration) and the addition of an aggressive agitation method after digestion (i.e. probe sonication for a short duration), the GCs of many plant species can be separated from neighboring cells with minimal damage.

In this work, we present a method for isolating intact GCs, retaining their cell walls, from *Vicia faba*. Building upon protocols used to protoplast GCs [24], our protocol uses a combination of blending and filtering, enzyme digestion, and probe sonication. The most unique, but critical, step in this process is the use of probe sonication, which we believe to work due to the increased contents of cellulose in the cell wall of GCs in comparison to other plant cell types. Characterization techniques used to verify GC isolation and integrity include optical microscopy and fluorescent confocal microscopy with propidium iodide (PI) staining. We are optimistic that the detailed procedures described here will enable quantitative compositional assays of GCs, catalyzing exploration into the composition of GC walls among plant biologists.

## Materials and methods

### Materials

Enzymes were purchased from Sigma-Aldrich (St. Louis, USA) and included pectolyase from Aspergillus japonicus, Driselase™ Basidiomycetes sp., and Cellulysin^®^ cellulase Trichoderma viride.

Filtering materials included 50 micron nylon mesh strainers purchased from Ted Pella Inc. (Redding, USA) and a 210 micron nylon mesh strainer (33% open area) purchased from Elko Filtering Co. (Tamarac, USA). Embroidery hoops were purchased from Amazon Inc. (Seattle, USA). The embroidery hoops were used for supporting the mesh strainers and were assembled following Jalakas *et al*.’s bio-protocol, but with the mesh not tensioned to create a funnel shape [25].

### Plant growth

Faba Beans Organics *Vicia faba* seeds were purchased from West Coast Seeds. *Vicia faba* were planted in potting mix (25% bark, 25% pumice, and 50% peat) and grown in the University of British Columbia’s (UBC) Greenhouse (Vancouver, Canada) during the winter at an average temperature of 23.5°C, 59 RH%, and natural light conditions. The plants were top-watered every three days.

### Isolation of guard cells

**Fig 2:**
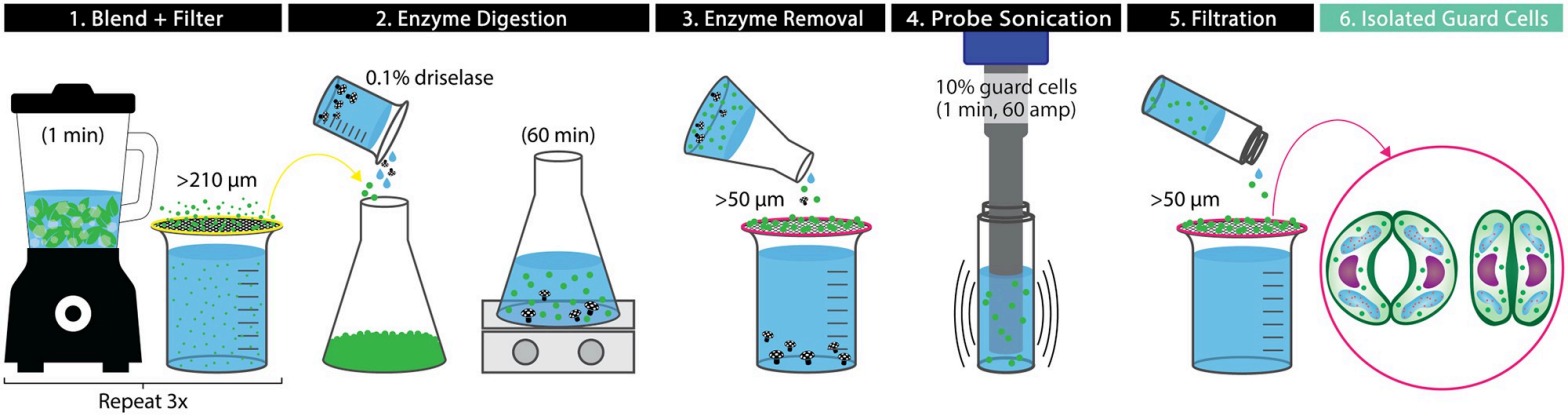
GC isolation process. A protocol for isolating guard cells from leaves, including: (1) blending and filtering through a 210 μm mesh filter three times, (2) enzyme digestion for 60 min using 0.1% Driselase, (3) enzyme removal by rinsing through a 50 μm filter, (4) agitation through probe sonication for one minute at 60 amps, (5) further filtration through a 50 μm filter to remove any ruptured cells, and (6) resulting in isolated guard cells.

This GC isolation protocol is adapted and built upon from the following procedures Pandey *et al*. [16], Kruse *et al*. [10], Yao *et al*. [26], and Jalakas *et al*. [25]. An overview of the protocol can be seen in Fig 2. In order to isolate GCs with their cell walls still intact, the following variables were adjusted: plant source, enzyme source, enzyme digestion time, sonication time, and sonication amplitude.

100 healthy, green leaves (approximately 35 g) were harvested at approximately 6–8 weeks old, after leaves had fully expanded and before flowers began to bloom, from various places on the plant and used the same day for GC isolation. The size of leaves and their surface areas are shown in S1 Table in S1 File and the methods for taking these measurements is explained in the characterization section. If leaves are not similar in size or at a different developmental stage when harvested, this may impact iGC yield [8], in which case the number of leaves [25], blending time [10, 14], enzyme digestion time [26], or other variables may have to be varied.

GCs were isolated by blending and filtering three times, using enzyme digestion, and then probe sonication. For blending and filtration, leaves, 250 mL RO water, and a hand full of crushed ice were added to a Black & Decker 10-speed Blender (New Britain, USA) and blending on speed setting 10 for one minute. The blended leaf mixture was filtered through a 210 μm nylon mesh strainer to remove mesophyll cells and rinsed with RO water until the foam from blending was mostly gone [26]. Blended material larger than 210 μm was scraped off of the mesh strainer with a metal spatula and put back in the blender with RO water and ice. This was repeated two more times, for a total of three times. After, the material on top of the nylon mesh strainer was lightly dried by placing paper towels underneath the strainer to wick water away. This material was then scraped off of the strainer and put in a dish, in preparation for the following isolation steps. At this stage, this material does not contain isolated GCs (iGCs), but rather a mix of plant cells that are still attached to one another and will be referred to as ‘cells’ throughout the remainder of the isolation process (i.e. until the stage in which iGCs are obtained).

For enzyme digestion, 0.5% (w/v) stock solutions of Driselase, pectolyase, and cellulase were prepared with RO water. In a flask, 20 mL of RO water, 5 mL of stock solution, 10 g of cells, and a magnetic stir bar were added and stirred for 1 hour in ambient conditions.

Using a plastic pipette, samples were removed at 30 and 60 minutes to determine the optimal digestion time. The pipette tip was cut to increase the opening diameter and reduce the shear stress on the cells [16]. After, the solution was filtered through a 50 μm mesh strainer, leaving cells in the strainer, and rinsed with RO water squirt bottle to agitate cells. The cells were scraped out of the strainer into a plastic Petri dish. Filtering the cells, helped in removing any residual cells that were not GCs.

After filtering, 0.7 g of cells and 7 mL of RO water were added to 7.4 mL glass vials. The solution was then probe sonicated for 60 seconds at 60 amps and filtered through a 50 μm mesh strainer, with the residue containing the iGCs. The iGCs were then stored in the glass vials at 3°C in the dark at a 10% (w/v) cell-RO water concentration.

### Characterization techniques

#### Isolated guard cell yield

iGC yields were determined by calculating the dry weight percentages of remaining plant cells after isolation. Measurements were taken on the same day that leaves were collected and GCs were isolated. First, the harvested leaves were sorted by size (small, medium, and large) into approximately three equal piles. In order to document the size of leaves used to isolate GCs, leaf surface areas were measured using pixel counts in ImageJ (S1 Table in S1 File) [27]. However, surface area measurements were not used in calculating the final yield of iGCs.

To calculate the final percentage of iGCs, dry weights, from three different isolation experiments, for the initial leaves and final iGCs were used. Dry weight of the leaves was determined by first measuring the wet weight of the three piles of leaves with a SECURA324–1S analytical balance. Their moisture content was then measured with a Mettler Toldeo Halogen Moisture Analyzer HC103 (115V). A standard drying program was run at 60°C with 5 (1 mg/140 s) switch-off criterion. Using the wet weight and moisture content of each pile, the dry weight was calculated using Eq 1. Similar to the dry weight of the leaves, the dry weight of the iGCs was determined by first measuring the wet weight and then the moisture content.

$$\begin{array}{c} Mass\;dried\;\left(g\right)=Mass\;original-\frac{\left(Moisture\;level\;x\;Mass\;original\right)}{100} \end{array}$$ (1)

#### Optical microscopy

Optical images were taken immediately after samples were prepared using a Nikon ECLIPSE LV100N POL microscope fitted with a Nikon DS-Ri2 camera.

#### Fluorescent microscopy with propidium iodide stain

To visualize cell membrane permeability, propidium iodide (PI) staining was performed following a modified version of Lucas *et al*.’s procedure [28]. 30 μL of fresh iGCs were stained for 5 min in 30 μL of aqueous solution containing 0.2 μg/μL of PI. 20 μL of iGCs were then mounted on a cover slip, with a glass slide placed on top, and sealed with clear nail polish to prevent evaporation.

**Fig 3:**
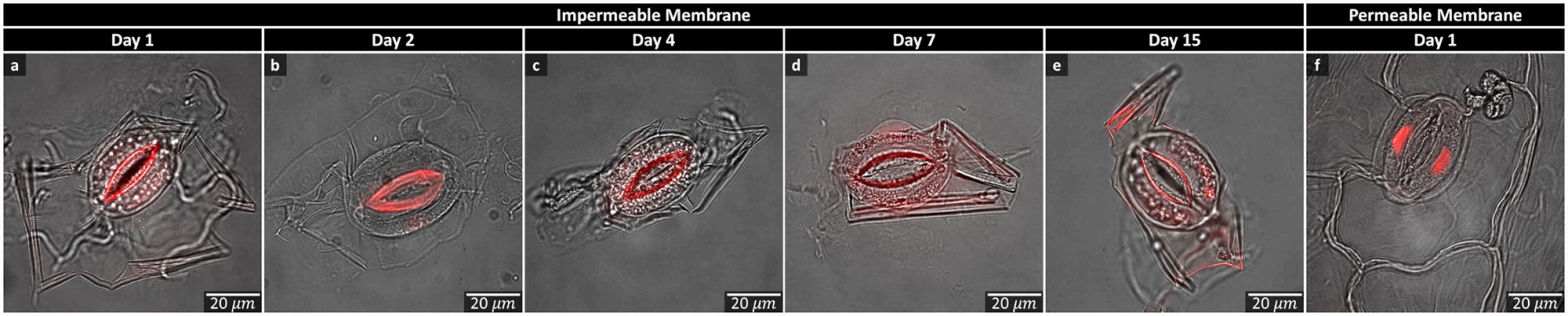
Cell membrane integrity/permeability. Confocal microscope images of *Vicia faba* guard cells (GCs) stained using propidium iodide. (a-e) show isolated GCs that are 1–15 days old. While the cell membranes appear impermeable, due to staining of the cell wall, the chloroplasts appear fragmented and granular. (f) shows a dead GC fixed in ethanol with the nuclei uniformly stained and demonstrating permeability of the membrane.

The slides prepared for Fig 3 were viewed with a ×63 oil objective (Hamamatsu 9100–02 CCD camera, 30.6 frames/sec) using a Leica DMi8 inverted microscope with a Yokogawa CSU-X1 spinning disk scan head. PI fluorescence was examined with a 595-nm laser. Images were captured using Improvision Volocity software and managed with Fiji (also referred to as ImageJ) and Adobe Illustrator.

## Results

In conventional approaches to GC isolation, the cell wall is typically removed through a process known as protoplasting, leaving only the cell membrane and its intracellular contents. In this work, we have presented a method for isolating GCs with their cell walls still intact using *Vicia faba* leaves, which is highlighted throughout the Figures in green text boxes. To achieve this, we modified traditional protoplasting procedures of blending, filtering, and enzyme digestion by decreasing the enzyme digestion time and adding the additional step of probe sonication. While it is anticipated that this protocol could work for all plants that protoplasting works for, further refinement may be necessary in adapting this procedure to other plants.

### Purity of guard cells at different stages of the isolation process

**Fig 4:**
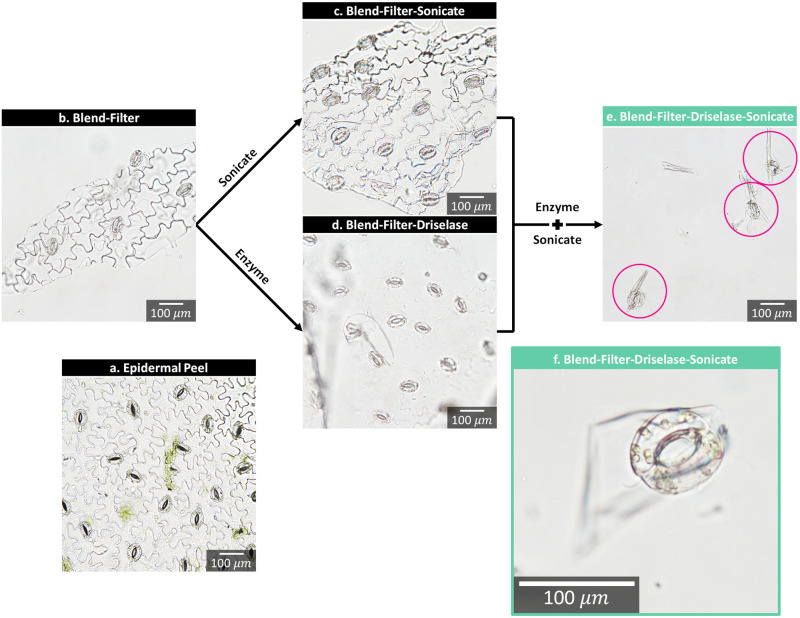
Guard cell isolation techniques. Optical microscope images of different methods used in attempting to isolate guard cells (GCs) from *Vicia faba*. The processes include: (a) epidermal peeling; (b) blending and filtering; (c) blending, filtering, and probe sonication; (d) blending, filtering, and digesting with Driselase; and (e) blending, filtering, digesting with Driselase, filtering (50 μm), and probe sonication. (f) An isolated GC of greater magnification, showing fully intact, spherical chloroplasts is also shown. Isolated GCs are circled. In all cases, except where specified, the filter size was 210 μm, enzyme digestion time was 60 min, and probe sonication was performed at 60 amps for 60 sec.

Optical microscopy was employed to capture images of *Vicia faba* at different stages of the iGC process and compared to an epidermal peel (Fig 4). All images (Fig 4a–4d), except the final iGC stage (Fig 4e and 4f), visually appeared similar, with the primary contaminates including epidermal cells, chloroplasts, and vascular tissue. Mesophyll cells were no longer present and chloroplasts appeared as clusters, which is a sign of broken mesophyll cells [10].

The stages involving blending and filtering, coupled with probe sonication, yielded results similar to the epidermal peel (Fig 4a), but with fragmented epidermal pieces (Fig 4b and 4c). When the process incorporated blending, filtering, and Driselase, it was visibly apparent that epidermal cells were beginning to change into spheres (Fig 4d), suggesting that the shorter enzymatic digestion time was sufficient to partially digest the cell wall of epidermal cells, while minimizing damage to the GC’s cell walls. Thus, blending and filtering in conjunction with an enzyme and sonication was necessary.

Images of iGCs are depicted in Fig 4e and 4f and S1 Fig in S1 File. Chloroplasts appeared spherical, rather than fragmented and granular, indicating minimal damage to the GCs using this isolation method. Minor residual contaminates were present and included epidermal fragments, which remained attached to iGCs, and vascular tissues. If necessary, contaminates can be further removed using a shaking water bath, centrifugation, and filtration [26, 29]. However, for most applications, further purification is not necessary. Following this isolation procedure, without further purification, iGCs made up 66.875 ± 7.009% of the starting leaf mass, with the percentage calculated using dry weights for the initial leaf mass and final iGC mass.

### Varying the guard cell isolation procedure

We started with a plant source survey, with no plants containing subsidiary cells, identifying which leaf source would be the easiest to see under an optical microscope and separate the epidermis from. It is known that leaves that are easier to peel by hand result in less contaminants when proplasting [16], so we began by looking into plant sources with existing proplasting protocols. Additionally, we looked into how different plant sources looked under the optical microscope after blending and filtering. It was interesting to note during these stages that for some plant sources the cutting action of blending leaves has a greater tendency to cut through epidermal cells, while leaving GCs intact (S2 Fig in S1 File). Specifically, this was observed for the *Polystichum munitum*, commonly referred to as Western Sword Fern, in which GCs cut into quarters from blending and filtering.

An explanation for the distinct cutting pattern of the Wester Sword Fern’s GCs might stem from how these ferns GCs are stiffened and strengthened. Unlike most plants surveyed, mainly comprising of angiosperms, ferns possess lignified polar end-walls, and exhibit higher cellulose concentrations at the central stomatal region. In contrast, angiosperms, such as *Arabidopsis*, typically have cellulose microfibrils in place of lignified cell walls at their polar end-walls and lack the higher concentration of cellulose at the stomata’s center, which corresponds to a higher stress in this region [23]. This variance in GC structure might also explain why the GCs of *Vicia faba*, an angiosperm, did not exhibit cutting and could be further used to hypothesize whether GCs of other plant species would cut. Additionally, *Vicia faba* proved to be the most suitable plant for separating the epidermal layer and easily seeing in an optical microscope. Therefore, the focus shifted to using *Vicia faba* as the plant source.

The effect of enzyme digestion on GC isolation was then tested. Three different enzymes were used after blending and filtering, including Driselase, pectolyase, and cellulase. Initially, we had anticipated pectolyase to be sufficient in separating cells by exploring the fact that cells are held together by pectin, as Bellinger *et al*. had done [30], but this did not prove to be the case for *Vicia faba*. Of these three enzymes, only Driselase successfully yielded iGCs, pectolyase and cellulase showed similar results (S3 Fig in S1 File). We also experimented with enzyme digestion time, expecting too short a duration to result in a lack of cell separation and too long resulting in individual cells falling apart [30]. Initially, we tried 60 min, which yielded iGCs, but later found 30 min to also be sufficient (S4 Fig in S1 File).

We did not look into varying the time and amplitude of probe sonication. However, varying these paramaters, along with any of the other variables mentioned could aid in a more pure yield or in GC isolation from different plant sources.

### Assessment of cell membrane permeability

Fluorescent confocal microscopy was implemented to look at cell membrane permeability using PI. We took samples of the iGCs over a period of 15 days and found the membrane to remain impermeable during this time (Fig 3a–3e). In comparison, we fixed an epidermal peel using ethanol, which resulted in uniformly stained nuclei and demonstrated permeability of the membrane (Fig 3f). In conclusion, our method of isolating GCs resulted in intact cell walls and membranes that remained impermeable to PI.

According to Chitrakar *et al*., PI staining can also be used for cell viability assays, staining the cell walls of viable cells [31]. Due to the membranes remaining impermeable, our results suggest viability of the iGCs and potential for them to maintain function (i.e. the ability to open and close) after isolation. However, further GC viability and physiological response experiments need to be carried out in future work to confirm this.

## Discussion, conclusions, & future work

In conclusion, this study has successfully developed a novel method for isolating GCs from *Vicia faba* while preserving their cell walls. This approach uses blending, filtering, enzyme digestion, and, most critically, probe sonication. To verify that GCs were isolated and their integrity, we employed optical microscopy and fluorescent confocal microscopy, stained with propidium iodide (PI). The results support our initial hypothesis that the refined protocol could serve as a catalyst for plant biologists aiming to conduct quantitative compositional assays of GC walls. Therefore, this work sets the stage for future explorations into the structure and chemical composition of GC walls.

In future work, refining the protocol to completely remove contaminates found among the iGCs would be of interest. Quantitative evaluations comparing the yield of iGCs versus contaminants using cell counting techniques could offer additional insights. Additionally, investigating plant species with higher GC densities—commonly found in xeromorphic leaves as opposed to mesomorphic or hygromorphic leaves—may increase iGC yields [13].

A key question warranting exploration is whether iGCs retain their responsiveness to external stimuli in the absence of neighboring epidermal cells, whose role in stomatal movement through changes in turgor or osmotic pressure remains largely uninvestigated [23]. Potential applications also extend to biobased materials [32]. Leveraging the unique structural characteristics and cellulose abundance in GC cell walls, it may be feasible to engineer iGCs that retain responsiveness post-cell death. For example, Gao *et al*. produced biobased helical magnetic microswimmers using the chiral crystalline cellulose of *Agapanthus africanus* [33]. Similarly, the cellulose structure in iGCs could serve as a reinforcing element (i.e. rebar) within the cell wall, while modifications to the intercellular components could enable the GCs to respond to external stimuli. If this modification were in the form of a hydrogel, it could aid in the preservation of the GC, which could further help further our compositional knowledge of GCs.

## Supporting information

S1 File (ZIP)
