# Supplementary material for: Preparation of isolated guard cells, containing cell walls, from Vicia faba
Source: PLoS One. 2024 Mar 21;19(3):e0299810. doi: 10.1371/journal.pone.0299810 (PMC10957180; doi:10.1371/journal.pone.0299810)
Supplement: S1 File — (ZIP) [file pone.0299810.s001.zip › S1_File.pdf]

Supporting Information: Preparation of isolated guard cells, containing cell walls, from *Vicia faba*

Sara K. Fleetwood[1], Maya Kleiman[2*], E. Johan Foster[1*]

[1] Department of Chemical and Biological Engineering, University of British Columbia, Vancouver, British Columbia, Canada

[2] Plant Sciences Institute, Agricultural Research Organization (Volcani Center), Rishon LeZiyyon, Israel

* Corresponding author

E-mail: mayakl@volcani.agri.gov.il (MK), johan.foster@ubc.ca (EF)

Supporting information

S1 Table. ***Vicia faba* dry weight & leaf size.** Average dry weight (g) and surface area (cm^2) per leaf of freshly picked *Vicia faba* leaves, before guard cell isolation and after being sorted into three sizes: small, medium, and large [1].

leaf size	dry weight (g)	surface area (cm^2)
Small	0.2 ± 0.1	23.5 ± 3.1
Medium	0.3 ± 0.0	39.4 ± 4.9
Large	0.5 ± 0.1	60.5 ± 3.9

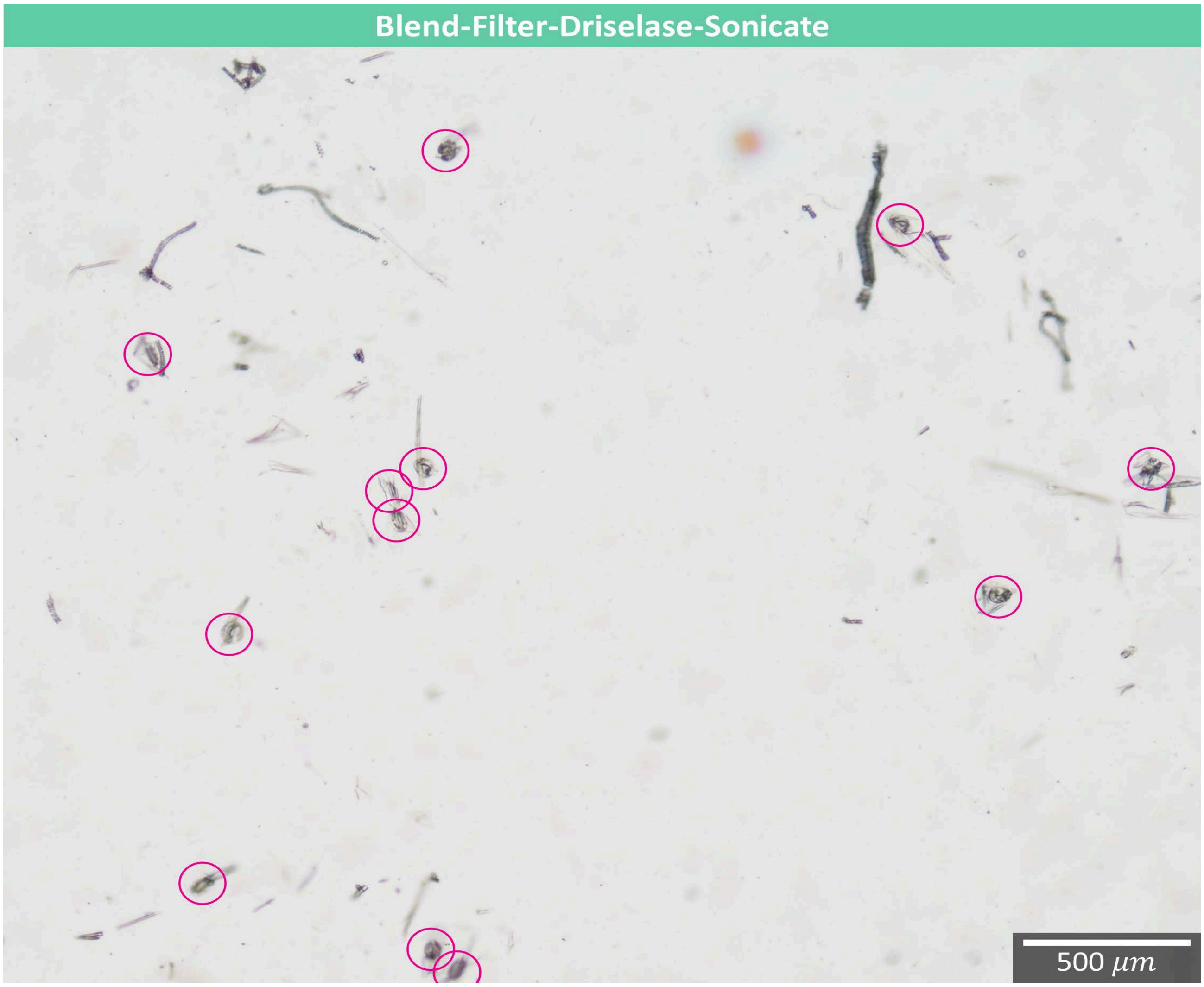

S1 Fig. Isolated guard cells. Optical microscope image of isolated guard cells (circled) from *Vicia faba*. The process includes blending, filtering (210 µm), digesting with Driselase for 60 min, filtering (50 µm), and probe sonication (60 amps for 60 sec).

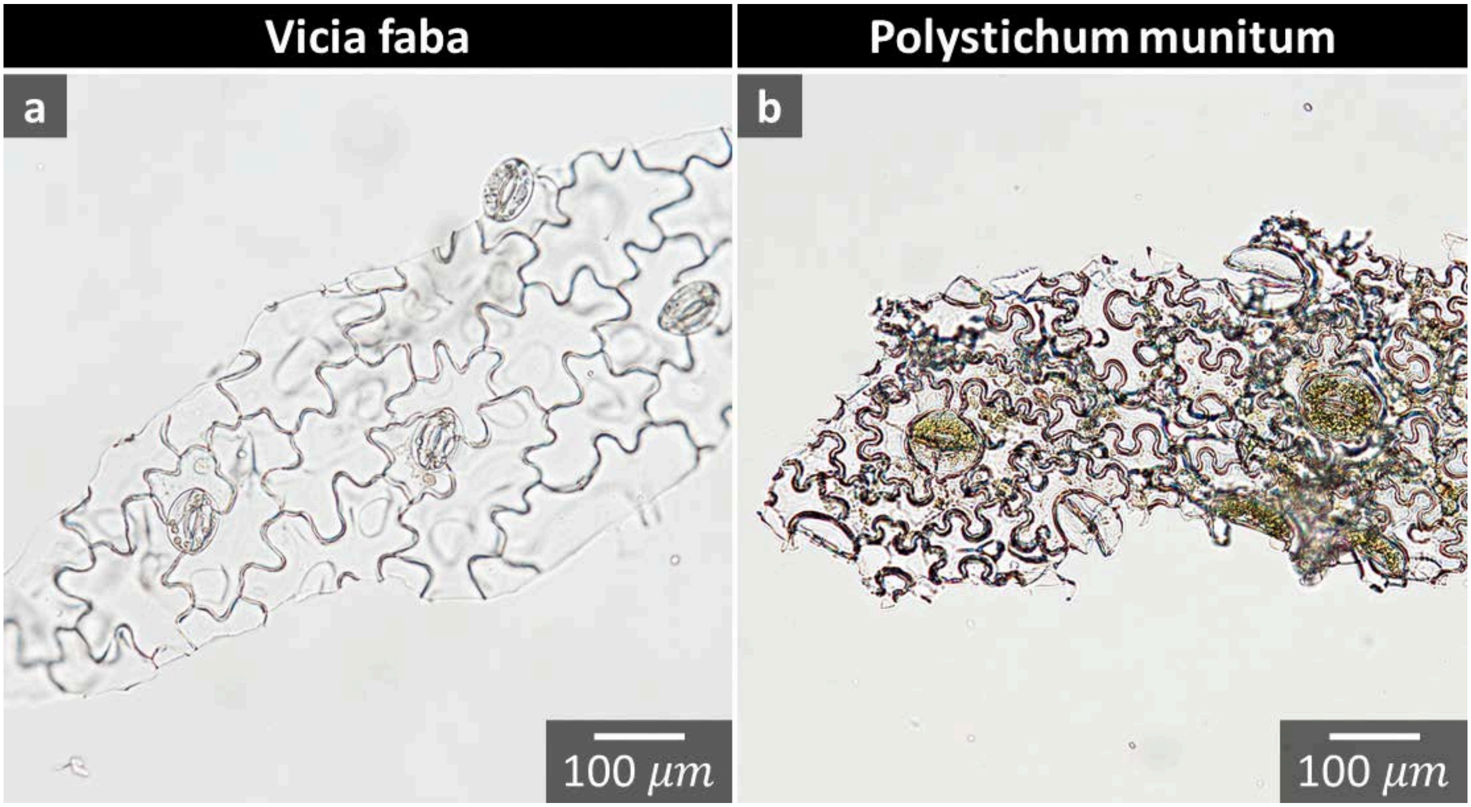

S2 Fig. Plant source. Optical microscope image of blended and filtered leaves showing the cutting of epidermal cells: (a) while leaving guard cells intact for *Vicia faba* and (b) guard cells fracturing into quarters for *Polystichum munitum*.

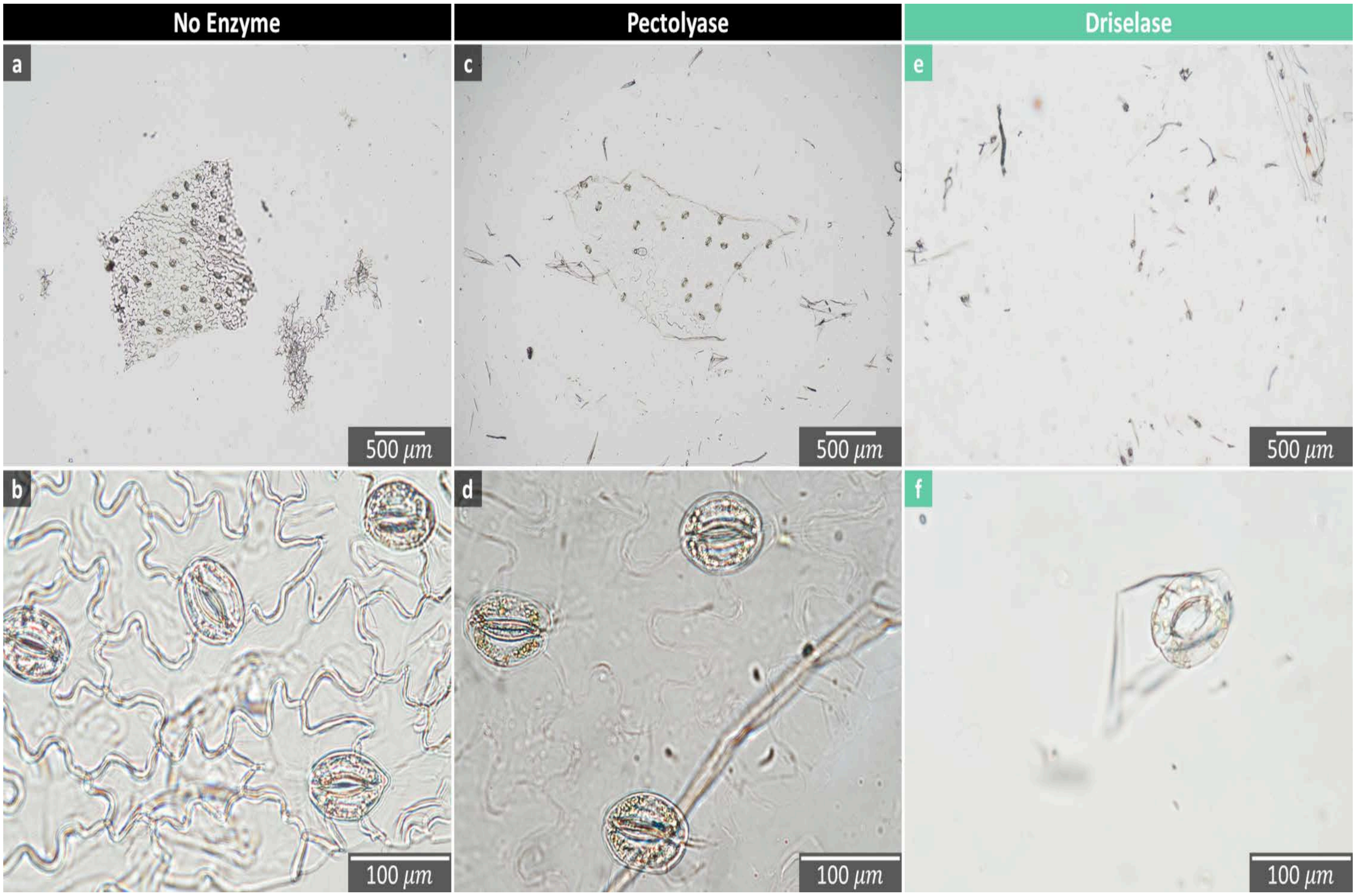

S3 Fig. Enzymes. Optical microscope images comparing different enzymes on guard cell isolation of *Vicia faba* blended, filtered at >210um, enzyme added and stirred for 60 min, filtered at >30 µm, and probe sonicated for 60 sec at 60 amps. The plants used included: (a-b) no enzyme, (c-d) pectolyase, and (e-f) Driselase.

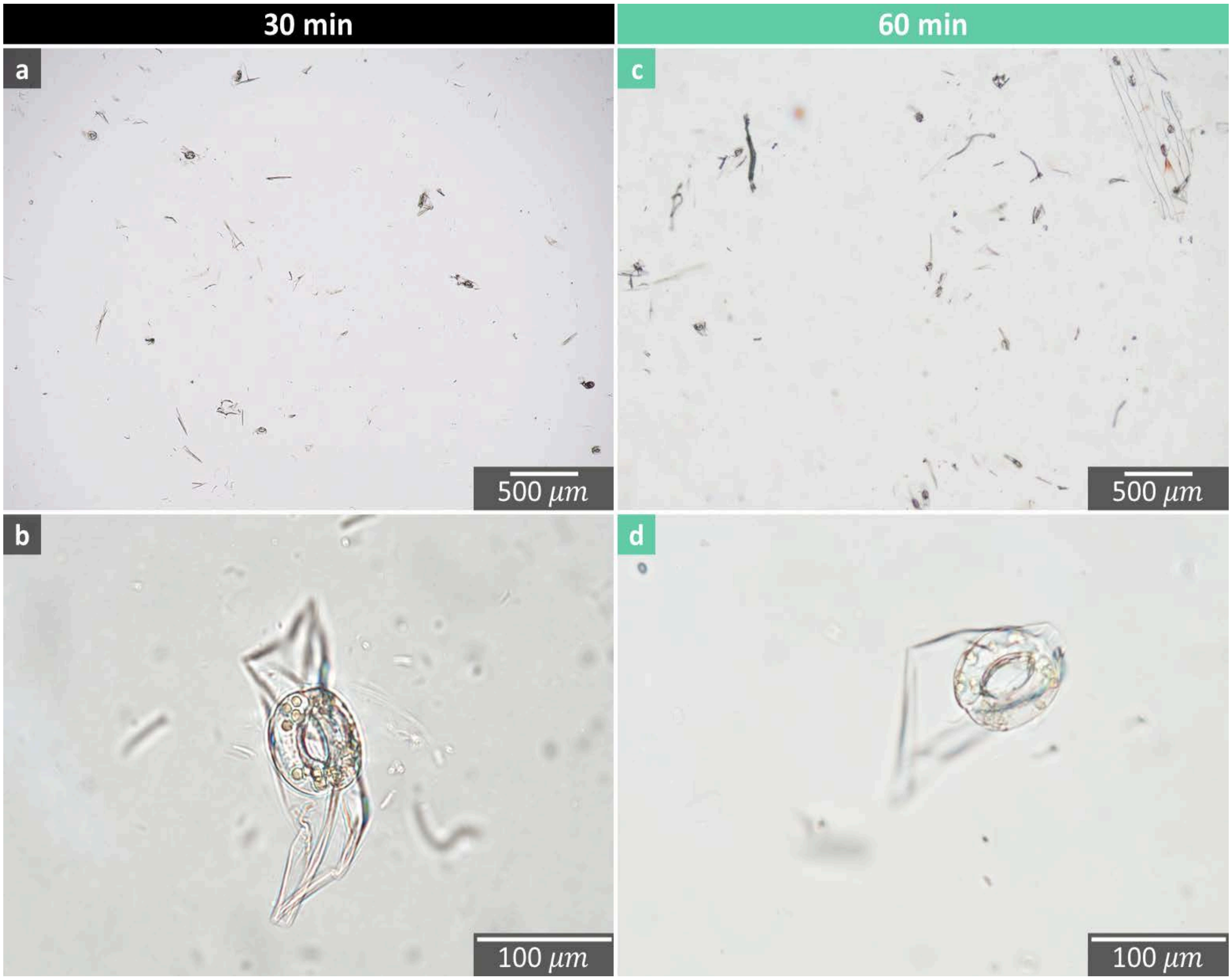

S4 Fig. Blending time. Optical microscope images comparing and summarizing different enzyme digestions times on guard cell isolation of *Vicia faba*. The digestion times included: (a-b) 30 min and (c-d) 60 min.

References

1. Fleetwood S, Kleiman M, Foster J. S1 Table Raw Data - Vicia faba dry weight & leaf size [Dataset]; 2024 [cited 2024 Feb 29]. Database: Dryad [Internet]. Available from: `https://doi.org/10.5061/dryad.hx3ffbgn2`.
